# Paraneoplastic limbic encephalitis presenting as a neurological emergency: a case report

**DOI:** 10.1186/1752-1947-4-95

**Published:** 2010-03-24

**Authors:** Zongqi Xia, Brijesh P Mehta, Allan H Ropper, Santosh Kesari

**Affiliations:** 1 Department of Neurology, Brigham and Women's Hospital, Boston, MA, USA; 2 Department of Neurology, Massachusetts General Hospital, Boston, MA, USA; 3 Department of Neurosciences, Moores UCSD Cancer Center, University of California, San Diego, La Jolla, CA, USA; 4 Dana-Farber Cancer Institute, Center for Neuro-Oncology, Boston, MA, USA

## Abstract

**Introduction:**

Paraneoplastic limbic encephalitis remains a challenging clinical diagnosis with poor outcome if it is not recognized and treated early in the course of the disease.

**Case Presentation:**

A 65-year-old Caucasian woman presented with generalized tonic-clonic seizures and increasing confusion shortly after a lung biopsy that led to the diagnosis of small-cell lung cancer. She had a complicated hospital course, and had recurrent respiratory distress due to aspiration pneumonia, and fluctuating mental status and seizures that were refractory to anti-epileptic drug treatment. Routine laboratory testing, magnetic resonance imaging of the brain, electroencephalogram, lumbar puncture, serum and cerebrospinal fluid tests for paraneoplastic antibodies, and chest computed tomography were performed on our patient. The diagnosis was paraneoplastic limbic encephalitis in the setting of small-cell lung cancer with positive N-type voltage-gated calcium channel antibody titer. Anti-epileptic drugs for seizures, chemotherapy for small-cell lung cancer, and intravenous immunoglobulin and steroids for paraneoplastic limbic encephalitis led to a resolution of her seizures and improved her mental status.

**Conclusion:**

Early recognition of paraneoplastic limbic encephalitis and prompt intervention with immune therapies at the onset of presentation will probably translate into more favorable neurological outcomes.

## Introduction

Paraneoplastic limbic encephalitis (PLE) was established as a distinct clinical and pathological entity by the British neuropathologist Corsellis and colleagues in 1968. They described three patients with bronchial carcinoma who had developed a subacute onset of memory loss and had displayed inflammatory and degenerative changes in the limbic region on post-mortem examination [1]. The current hypothesis for the pathogenesis of PLE implicates an autoimmune process involving antigens shared by tumor cells and neuronal cells in the mesial temporal and limbic structures, including cingulated gyrus, orbitofrontal cortex and mamillary bodies [2]. The most frequently associated neoplasm is small-cell lung cancer (SCLC), followed by germ cell tumor of the testis, breast cancer, Hodgkin's lymphoma, thymoma, and immature teratoma of the ovaries [3].

Dalmau and colleagues proposed the first criteria for establishing the diagnosis of PLE in a patient [3]. First, the clinical presentation must be a subacute onset of neurological (e.g. short-term memory loss, complex partial or generalized seizure) and psychiatric symptoms (e.g. depression, anxiety, irritability, sleep disturbance, paranoia or hallucination) with an insidious course. Second, neuropsychiatric symptoms must often precede the recognition of an underlying malignancy by up to four years, with a mean latency of three to five months. Third, the diagnostic evaluation must exclude other complications of cancer that might also cause limbic dysfunction (e.g. brain metastasis, metabolic and nutritional deficits, and adverse effects of chemotherapy or radiation therapy). Finally, the diagnostic evaluation should also reveal at least one of the following supporting evidences: (1) inflammatory changes in the cerebrospinal fluid (CSF), such as mild to moderate lymphocytic pleocytosis with fewer than 100 cells, mildly elevated proteins of <150 g/L with a high immunoglobulin G (IgG) index and the presence of oligoclonal IgG bands, but without any malignant cells in cytology; (2) one or both temporal lobe abnormalities on MRI such as hyperintense signals on T2-weighted or fluid-attenuated inversion recovery (FLAIR) sequences, atrophic temporal-limbic structures on T1-weighted images, typically without contrast-enhancement in the brain parenchyma or leptomeninges; (3) focal slowing or epileptiform activity in one or both temporal lobes on electroencephalography (EEG). Although temporal lobe seizures or status epilepticus is non-specific, its recognition is crucial to symptomatic management and patient stabilization. PLE is rare in patients with both normal EEG and normal brain MRI [4].

The list of differential diagnosis with similar clinical presentation includes viral encephalitis (e.g. herpes simplex virus [HSV]), lupus cerebritis, toxic and metabolic encephalopathies, multiple sclerosis, Hashimoto's encephalopathy, Wernicke's encephalopathy, neurosyphilis, primary vasculitis of the central nervous system, and leptomeningeal involvement of malignancy [2].

There is no evidence-based recommendation for the treatment of PLE. Current opinion favored applying a two-pronged approach for our patient with PLE, using a combination of tumor removal to eliminate the source of paraneoplastic onconeuronal antigens, and immune therapy (e.g. intravenous steroid, intravenous immunoglobulin (IVIG), or plasma exchange) to prevent further immune-mediated neuronal injury [2,5]. The prognosis for recovery in patients with PLE is poor if immune therapy is administered without concomitant treatment of the underlying malignancy [3]. Prompt initiation of immune therapy is probably associated with an improved overall outcome.

The prognosis is dependent upon the type of associated paraneoplastic onconeuronal antigen. In general, cytotoxic T-cell mediated process associated with intracellular antigens (e.g. Hu) is less responsive to the aforementioned two-pronged approach of tumor removal and immune therapy than immune process associated with cell surface antigens, and thus carries a worse neurological outcome [6].

## Case Presentation

A 65-year-old, right-handed Caucasian woman was transferred from a local hospital to our neurological intensive care unit (NICU) after a witnessed generalized tonic-clonic seizure. She had hypertension, diabetes mellitus, dyslipidemia, chronic obstructive pulmonary disorder, a history of heavy cigarette smoking and a long-standing but well-controlled bipolar disorder. Three weeks before the seizure, biopsy of a right hilar mass via mediastinoscopy confirmed the diagnosis of SCLC. Treatment for the cancer had not been initiated. Shortly after the biopsy, her family found her to be intermittently confused. Over the following two days, she developed fever, dizziness, vomiting, poor appetite and progressive shortness of breath. Confusion worsened to the point that she could no longer recognize her family and became non-interactive. She remained lethargic and confused despite treatment with ceftriaxone and azithromycin for right lower lobe pneumonia. Investigations and results at the local hospital included: (1) CSF analysis showed 28 white blood cells with 99% lymphocytes, glucose 122 g/L, protein 36 g/L, no organisms on Gram stain and no growth from bacterial culture; (2) MRI of the brain showed T2 and FLAIR hyperintensities in both mesial temporal regions without contrast enhancement, diffusion or susceptibility changes; (3) EEG demonstrated background slowing with focal sharp and slow discharges in the right mid-temporal and right posterior temporal region. Empiric treatment with intravenous acyclovir was initiated while HSV polymerase chain reaction (PCR) study from CSF was sent for analysis. On her seventh day at the local hospital, she was observed having a generalized tonic-clonic seizure for which she received intravenous fosphenytoin. She was intubated and transferred to our NICU.

On examination, she was unresponsive to verbal or noxious stimuli. Her pupils were restricted from previous cataract procedures. There was no gaze preference. Corneal, vestibular-ocular and gag reflexes were intact. Her tone was normal. She made no purposeful withdrawal on the left side. A Babinski maneuver elicited a plantar response from both feet.

**Figure 1:**
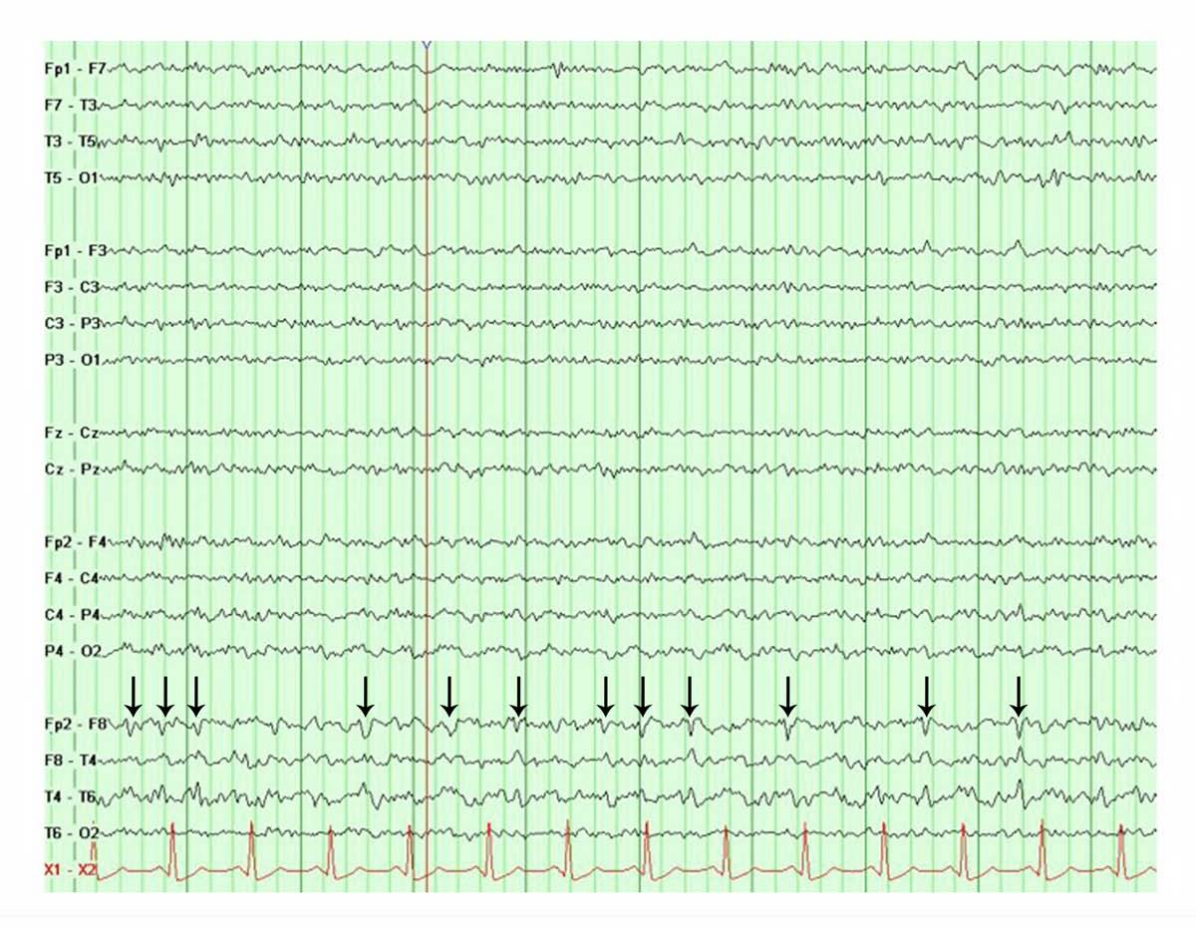
**Electroencephalography (low frequency 10 Hz, high frequency 70 Hz) showing epileptiform activity is comprised of bilateral independent periodic lateralized epileptiform discharges (bi-PLEDs) in both temporal lobes with a right-sided predominance (arrows)**.

**Figure 2:**
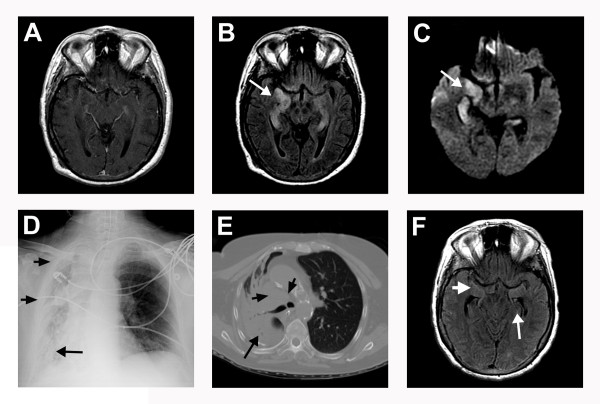
**Magnetic resonance imaging (MRI) of the brain**. (A) T1-post-gadolinium sequence with no enhancement. (B) T2-fluid-attenuated inversion recovery (FLAIR) sequence with hyperintensities in both mesial temporal lobes (arrow). (C) Diffusion-weighted images with T2 shine-through in the right temporal lobe and hippocampus (arrow). (D) Chest X-ray with right lower lobe infiltrates consistent with post-obstructive pneumonia (long arrow), complete opacity of the right middle and upper lobes due to atelectasis from small-cell lung cancer (short arrows). (E) Chest computed tomography scan with contrast of mediastinal and hilar lymphadenopathy (short arrows) and a loculated right lower lobe pleural effusion (long arrows). (F) MRI of the brain 6 months after initial neurological presentation with temporal horn enlargement (short arrow), hippocampal atrophy (long arrow) and resolution of T2-FLAIR hyperintensities.

She had a second, brief, witnessed generalized tonic-clonic seizure with left gaze deviation and left head turning. An EEG demonstrated electrographic seizures consisting of bilateral independent periodic lateralized epileptiform discharges (bi-PLEDs) in both temporal lobes with right-sided predominance (Figure 1). Intravenous lorazepam was given and phenytoin was reloaded. Levetiracetam and topiramate were subsequently added in increasing doses (up to maximum dosages) to treat persistent electrographic seizures. Repeat CSF analysis again demonstrated mild pleocytosis with lymphocytic predominance (23 white blood cells with 94% lymphocytes) but was otherwise normal. CSF cytology did not show any malignant cells. Empiric treatment with acyclovir for HSV encephalitis continued until a second negative CSF HSV PCR returned. Epstein-Barr virus (EBV), cytomegalovirus (CMV), varicella zoster virus (VZV) and human herpesvirus 6 (HHV-6) were all negative. Repeat MRI of the brain showed T2-FLAIR hyperintensities in both mesial temporal lobes without restricted diffusion or post-gadolinium enhancement (Figures 2A-C). Chest X-ray showed post-obstructive pneumonia in the right lower lobe with collapse of the right middle and upper lobes due to compressive atelectasis from the lung cancer (Figure 2D). Computed tomography (CT) scan of the chest demonstrated mediastinal and hilar lymphadenopathy and pleural effusion in addition to a loculated right lower lobe (Figure 2E). Her lung cancer was not amenable to resection. She continued to receive broad-spectrum antibiotics for pneumonia, and chemotherapy for SCLC with carboplatin and etoposide was commenced.

Serum contained markedly elevated anti-N-type voltage-gated calcium channel (VGCC) antibody titer of 0.42 nmol/L (normal value < 0.03) and mildly elevated anti-P/Q-type VGCC antibody titer of 0.04 nmol/L (normal value < 0.02). No other paraneoplastic antibody (including binding, ganglionic and striational acetycholine [Ach] receptor antibodies, Purjinke cell antibodies type 1 [PCA-1 or anti-Yo], PCA-2, PCA-Tr [anti-Tr, immune response marker for Hodgkin's lymphoma], anti-neuronal nuclear antibodies type 1 [ANNA-1 or aniti-Hu], ANNA-2 [anti-Ri], ANNA-3, anti-Ma1, anti-Ta, collapsin response-mediator protein-5 [CRMP-5 or CV2], amphiphysin, anti-glial/neuronal nuclear antibody, Type 1 AGNA-1) was detected. Voltage-gated potassium channel (VGKC), N-methyl-D-aspartate receptor (NMDAR) and glutamic acid decarboxylase (GAD) antibodies were not sent. Our patient received IVIG (0.4 mg/kg daily for five days) and 1 g of Solu-medrol (methylprednisolone) daily for three days, followed by tapering doses of prednisone over four weeks. Long-term monitoring (LTM) EEG showed gradual resolution of epileptiform activities only after initiation of immune therapy, despite being on multiple anti-epileptic drugs.

Two months after the onset of her neurological illness, she was following commands and had become conversant but she still displayed cognitive impairment and still suffered episodes of delirium. Our patient had multiple subsequent hospitalizations for aspiration pneumonia and seizures that were refractory to anti-epileptic drug adjustments and only responsive to IVIG and steroids on a monthly basis. MRI six months later showed resolution of T2-FLAIR hyperintensities (Figure 2F). However, she continued to have severe short-term memory deficits. She died eight months after the initial presentation due to progressive lung cancer.

## Discussion

Although the association between PLE and auto-antibodies against neuronal antigens (nuclear, cytoplasmic or ion channel) is well-known [3], there has been no definitive proof of any specific cause-effect relationship between the presence of antibodies and the development of clinical syndrome [7]. Onconeuronal auto-antibodies have been found in cancer patients without apparent paraneoplastic neurological syndrome [8], and they have been undetectable in a subpopulation of symptomatic patients [9]. Since onconeuronal auto-antibodies in PLE are best considered as biomarkers rather than as a proof of pathogenesis, the absence of paraneoplastic neuronal antibodies does not necessarily argue against PLE [4]. While the P/Q-type VGCC auto-antibody is known to be associated with Lambert-Eaton myasthenic syndrome, cerebellar degeneration and SCLC [10,11], the association with the N-type VGCC has not been well-described. In this case, the high titer of the N-type VGCC antibody in the setting of SCLC is insufficient to prove the auto-antibody was the cause of the neurological decline.

Although PLE is rare (with a slightly greater predominance among women), the disease is under-reported due to the difficulty in establishing the diagnosis [12]. Several factors render the diagnosis of PLE challenging. First, many other cancer-related complications, including brain metastases, toxic and metabolic encephalopathies, and adverse effects of cancer therapy, may have similar insidious neuropsychiatric presentations. Second, diseases other than PLE, particularly those of an infectious etiology such as HSV, share similar initial clinical features to PLE. Third, there is no "gold standard" method of establishing a diagnosis. The pathological findings of neuronal loss, perivascular inflammatory infiltrates, microglial activation, and reactive astrocytosis in the temporal and limbic structures are non-specific and do not establish a paraneoplastic etiology.

The unusual feature of our case of PLE is the clinical course of neuropsychiatric manifestation in relation to the discovery of the underlying malignancy. In a recent case report, a patient with refractory seizure, in the setting of testicular cancer and anti-Ma-2 antibodies, showed no evidence of memory decline or psychiatric symptoms prior to presentation [13]. Likewise in our case, there was no history of neuropsychiatric manifestation prior to the biopsy that led to the eventual diagnosis of SCLC. In fact, our patient presented with altered mental status and subsequent seizures only after the biopsy. This raised the intriguing possibility that perturbation of the tumor tissues might have accelerated an immune response to onconeuronal auto-antigens and inflammation-mediated neuronal injury. The pneumonia might have contributed initially, but even after treatment of the pneumonia, the neurological status of our patient continued to decline. Otherwise, the clinical picture in our case is consistent with the diagnosis of PLE, which is additionally supported by results from the CSF, MRI, EEG and paraneoplastic antibody studies.

In the early stages of the disease, PLE and HSV encephalitis share not only similar clinical presentation but also comparable MRI features. However, MRIs of patients with HSV encephalitis typically demonstrate more prominent gyral enhancement on T1-weighted post-gadolinium sequences. They also demonstrate more significant mass effects from cytotoxic edema on T2-weighted sequences, and more evident hemorrhages on susceptibility sequences [3,4]. Additionally, the presence of disproportionally elevated red blood cells in the CSF further raises the suspicion for HSV encephalitis. One caveat is that PCR for HSV from the CSF could be negative if the CSF is obtained within the first 72 hours after the onset of neurological symptoms, despite the otherwise high sensitivity and specificity of this method of detection [14].

In our case, HSV encephalitis clearly ranked high in the initial differential diagnosis, given the patient's seizure in the setting of fever and her history of altered mental status. In retrospect, post-obstructive pneumonia was the likely cause of the fever. Nevertheless, HSV encephalitis was not an unreasonable initial working diagnosis of equal possibility to PLE.

The median survival time after diagnosis of PLE ranges between 22 and 43 months [2]. The overall poor outcome of PLE likely stems from both the delay in recognition and treatment, and the resulting immune-mediated neuronal injury. Thus, we advocate the initiation of immune therapy as soon as a clinical suspicion of PLE is raised. Even with prompt treatment, irreversible neuronal damage and neuropsychiatric complications in our patient might have been unavoidable due to the delayed therapeutic effect of immune therapy in disorders of the central nervous system [7]. Long-term complications include recurrent seizures, anterograde amnesia, cognitive impairments and chronic progressive encephalopathy with associated cerebral atrophy, particularly involving the mesial temporal and limbic structures [15].

Early immune treatment is particularly important with the emergence of rituximab, an anti-CD20 antibody that depletes B-cells and potentially the source of paraneoplastic antibody production [16]. B-cells cross the blood-brain barrier and intrathecal production of antibodies correlates with clinical severity in paraneoplastic disease [17]. There is early evidence that rituximab is safe and may improve neurological outcomes, as seen in adjunct therapy in children with paraneoplastic opsoclonus-myoclonus syndrome [17], as well as in paraneoplastic syndromes related to intracellular auto-antigens [18]. Since these studies are small and non-randomized, we cannot make conclusive statements about the role of rituximab in the management of paraneoplastic diseases.

The greatest challenge in the management of patients with PLE is to promptly deliver immune therapy and cancer treatment. With SCLC, the definitive treatment for cancer is chemotherapy, since surgery rarely plays a role. To undergo chemotherapy, patients have to be medically stable. In the case of our patient, chemotherapy for SCLC did not commence until her pneumonia was better controlled. The predicament was that the pneumonia of our patient, which delayed chemotherapy in the first place, was probably the result of a post-obstructive etiology from the tumor burden of the lung cancer itself.

Another instructive lesson in our case is that immune therapy should have been initiated empirically at the onset of the presentation and continued until PLE could be excluded. This would be analogous to the approach of initiating empiric treatment with acyclovir until HSV encephalitis is definitively excluded. In both situations, the delay in treatment while waiting for supporting diagnostic studies, HSV PCR and paraneoplastic panels, respectively, could potentially lead to devastating neurological outcomes. The benefit of empiric treatment with acyclovir outweighs the risk of not pursuing therapy while awaiting the results of diagnostic tests. Likewise, we recommend that in most patients the risks of immune therapy (IVIG or plasma exchange, steroids) are low enough to warrant early empiric intervention if there is sufficient clinical suspicion of PLE. We recommend the following tests before proceeding with empirical therapy for PLE: MRI of the brain, CSF analysis for bacterial and viral agents (in particular HSV PCR), measurement of ammonia levels and thyroid-stimulating hormone (TSH), a metabolic and electrolyte panel, EEG, and a paraneoplastic panel of both blood and CSF.

## Conclusion

This case illustrates the complex diagnostic and treatment decisions that clinicians will need to make in a relatively short period of time. We wait far too often for the results of diagnostic testing and delay the definitive treatment for PLE. This causes patients to suffer devastating neurological sequalae. In the setting of known cancer, the decision to treat patients for paraneoplastic neurological syndrome should be empirically considered. Early intervention with immune therapies in patients at the onset of presentation will probably translate into more favorable neurological outcomes, but definitive practice parameters will have to be developed judiciously, especially with the development of potentially more effective targeted therapies such as rituximab.

## Consent

Written informed consent was obtained from the patient's next-of-kin of for publication of this case report and any accompanying images. A copy of the written consent is available for review by the Editor-in-Chief of this journal.

## Competing interests

The authors declare that they have no competing interests.

## Authors' contributions

ZX, BPM and SK took care of our patient. ZX drafted the initial manuscript. BPM created the figures and figure legends for the manuscript. ZX, AHR and SK revised the manuscript. All authors read and approved the final manuscript.
